# Toxicity and biodegradation of ibuprofen by *Bacillus thuringiensis* B1(2015b)

**DOI:** 10.1007/s11356-017-8372-3

**Published:** 2017-01-23

**Authors:** Ariel Marchlewicz, Urszula Guzik, Katarzyna Hupert-Kocurek, Agnieszka Nowak, Sylwia Wilczyńska, Danuta Wojcieszyńska

**Affiliations:** Department of Biochemistry, Faculty of Biology and Environmental Protection, University of Silesia in Katowice, Jagiellońska 28, 40-032 Katowice, Poland

**Keywords:** Biodegradation, Biotransformation, Ibuprofen, Toxicity, Cometabolism, *Bacillus*

## Abstract

In recent years, the increased intake of ibuprofen has resulted in the presence of the drug in the environment. This work presents results of a study on degradation of ibuprofen at 25 mg L^−1^ in the presence of glucose, as an additional carbon source by *Bacillus thuringiensis* B1(2015b). In the cometabolic system, the maximum specific growth rate of the bacterial strain was 0.07 ± 0.01 mg mL^−1^ h^−1^ and *K*_*sμ*_ 0.27 ± 0.15 mg L^−1^. The maximum specific ibuprofen removal rate and the value of the half-saturation constant were *q*_max_ = 0.24 ± 0.02 mg mL^−1^ h^−1^ and *K*_s_ = 2.12 ± 0.56 mg L^−1^, respectively. It has been suggested that monooxygenase and catechol 1,2-dioxygenase are involved in ibuprofen degradation by *B. thuringiensis* B1(2015b). Toxicity studies showed that *B. thuringiensis* B1(2015b) is more resistant to ibuprofen than other tested organisms. The EC50 of ibuprofen on the B1 strain is 809.3 mg L^−1^, and it is 1.5 times higher than the value of the microbial toxic concentration (MTC_avg_). The obtained results indicate that *B. thuringiensis* B1(2015b) could be a useful tool in biodegradation/bioremediation processes.

## Introduction

Every year, a great number of pharmaceutical compounds are consumed, and after their partial metabolism, they enter sewage treatment plants. However, the removal of these compounds is not efficient, and pharmaceuticals are still observed in the sewage treatment plant effluents, as well as in surface water, groundwater and even drinking water (Tambosi et al. 2010). Ibuprofen, one of the non-steroidal anti-inflammatory drugs, is the third most highly consumed pharmaceutical in the world. It has been detected in the environment at the ppt to ppb concentration range (Murdoch and Hay 2015).

Acute and chronic toxicity studies on daphnia and fish allowed the determination of the no observed effect concentration (NOEC) of ibuprofen, which was then used to calculate the predicted no-effect concentration (PNEC). Because the estimated MEC/PNEC (real risk) ratio for ibuprofen was over 1, a probable environmental risk was suggested (Bouissou-Schurtz et al. 2014). The hitherto toxic effects of ibuprofen on *Scenedesmus subspicatus*, *Pseudokirchneriella* *subcapitata*, *Daphnia magna*, *Daphnia longispina*, *Neocaridina denticulata*, *Danio rerio*, *Pimephales notatus*, *Oncorhynchus mykiss*, *Rutilus rutilus*, *Oreochromis niloticus*, *Oryzias latipes*, *Menidia beryllina* and *Mytilus galloprovinciali* as model organisms were evaluated (Brozinski et al. 2013; Fent et al. 2006; Flippin et al. 2007; Gonzalez-Naranjo and Boltes 2014; Gonzales-Rey and Bebianno 2012; Jeffries et al. 2015; Ragugnetti et al. 2011; Sung et al. 2014). Flippin et al. (2007) showed that ibuprofen increased the number of eggs with a simultaneous decrease in the number of fish spawning in Japanese medaka, *Oryzias latipes*. Exposition of *Mytilus galloprovincialis* on ibuprofen at an environmentally realistic concentration (250 ng L^−1^) confirmed the endocrine disruption effect of this drug. Moreover, in the presence of ibuprofen, the induction of antioxidative stress was observed. The activities of superoxidase dismutase, catalase, glutathione reductase and phase II glutathione *S*-transferase increased during 7 days of exposure. The increase in the lipid peroxidation level and, in consequence, membrane damage in the digestive gland of mussels were also observed (Gonzales-Rey and Bebianno 2012). Jeffries et al. (2015) suggested that a low concentration of ibuprofen, at 11.5 μg L^−1^, causes the downregulation of genes involved in skeletal development, aerobic respiration and immune function, whereas higher concentrations of ibuprofen may increase the expression of regulatory genes connected with the arachidonic acid metabolism pathway and immune genes involved in an inflammatory response. Considering all the above facts, it is important to develop the effective methods for the removal of ibuprofen from the environment.

The highest effectiveness of ibuprofen degradation is reached by using the physicochemical methods, especially advanced oxidation processes (AOP) (Gongora et al. 2016; Huang et al. 2015; Iovino et al. 2016). During the AOP, high reactive hydroxyl radicals are generated which initiate the oxidation of ibuprofen (Braz et al. 2014; Gongora et al. 2016; Li et al. 2015). Hydratropic acid, 4-ethylbenzaldehyde, 4-(1-carboxyethyl)benzoic acid, 1-(4-isobutylphenyl-1-ethanol, 2-[4-(1-hydroxy-2-methylpropyl)phenyl]propanoic acid, 1-isobutyl-4-vinylbenzene, 4-isobutylphenol, 4-acetylbenzoic acid and 4-isobutylacetophenon are formed in this process (Caviglioli et al. 2002; Ruggeri et al. 2013; Sabri et al. 2012). However, toxicity studies have shown that intermediates formed during the AOP treatment are more toxic than the primary compound (Braz et al. 2014; Huang et al. 2015; Quero-Pastor et al. 2014). For that reason, biological methods of ibuprofen degradation seem to be a good alternative. There is still little information on metabolites formed during the biodegradation of ibuprofen through activated sludge. However, Collado et al. (2012) observed the partial degradation of ibuprofen (<10%, 44% and 60% accounting for 10, 100 and 1000 μg/L of ibuprofen, respectively), with the simultaneous formation of intermediates: carboxyibuprofen and hydroxyibuprofen isomers. The latter were also detected by Quintana et al. (2005). So far, only four pure strains able to degrade ibuprofen have been described: *Nocardia* sp. NRRL 5646, *Sphingomonas* Ibu-2, *Variovorax* Ibu-1 and *Patulibacter* sp. I11 (Almeida et al. 2013; Chen and Rosazza 1994; Murdoch and Hay 2005; Murdoch and Hay 2013). Degradation of ibuprofen by *Patulibacter* sp. I11 was observed only in the presence of yeast extract and tryptone. The results of quantitative proteomic analysis suggest that acyl-CoA synthetase, a protein containing a Rieske-like (2Fe–2S) iron–sulphur cluster (dioxygenase-like protein), and enoyl–CoA hydratase/isomerase may be involved in the decomposition of ibuprofen (Almeida et al. 2013). Ibuprofenol and ibuprofenol acetate were detected during the degradation of ibuprofen by the lignolytic bacterium *Nocardia* sp. NRRL 5646 (Chen and Rosazza 1994).

Murdoch and Hay (2005, 2015) proposed the biodegradation pathway of ibuprofen in *Sphingomonas* Ibu-2 and *Variovorax* Ibu-1. In *Sphingomonas* Ibu-2, the degradation of ibuprofen occurs through isobutylcatechol as an intermediate. In the next step, isobutycatechol is cleaved to 5-formyl-2-hydroxy-7-methylocta-2,4-dienoic acid, transformed to 2-hydroxy-5-isobutylhexa-2,4-dienedioic acid. In this pathway, *meta*-cleavage enzymes are probably engaged (Murdoch and Hay 2005; Murdoch and Hay 2015). The participation of *meta*-ring fission enzymes in the biotransformation of ibuprofen by *Variovorax* Ibu-1 has also been suggested (Murdoch and Hay 2015).

In the light of the above-mentioned facts and the still limited knowledge on the degradation of ibuprofen by bacteria, it is very important to study the microbiological degradation of ibuprofen and its impact on the environment. Therefore, the aims of our work were to investigate the degradation of ibuprofen by the pure bacterial strain *Bacillus thuringiensis* B1(2015b) in a monosubstrate and cometabolic systems and to identify enzymes engaged in ibuprofen transformation. We have also evaluated the toxicity of ibuprofen to model organisms and *B. thuringiensis* B1(2015b) and determined the influence of ibuprofen on bacterial fatty acid profiles. To our knowledge, it is the first work which links studies leading to a better understanding of the ibuprofen biodegradation process and to the evaluation of the impact of this drug on the environment.

## Materials and methods

### Bacterial strain and growth conditions

*B. thuringiensis* B1(2015b) (Marchlewicz et al. 2016) was routinely cultivated in the nutrient broth composed of pancreatic digest of gelatin and beef extract (BBL® Nutrient Broth, Becton Dickinson, USA) at 30 °C and 130 rpm for 24 h. After this, cells were harvested by centrifugation (5000×*g* at 4 °C for 15 min), washed with a fresh sterile mineral salts medium (Greń et al. 2010, Cai et al. 2016) and used as inoculum.

### Adaptation of *B. thuringiensis* B1(2015b) to increasing concentration of ibuprofen

Studies on the degradation of ibuprofen were performed in 500-mL Erlenmeyer flasks containing 250 mL of mineral salts medium (Greń et al. 2010) inoculated with bacterial cells to a final optical density of about 0.8 (OD_600_) for the monosubstrate and 0.1 (OD_600_) for cometabolic systems. Ibuprofen was added to each flask to obtain an initial concentration of 1 and 5 mg L^−1^ for monosubstrate and cometabolic systems, respectively. If the complete degradation of ibuprofen was observed, a successive dose of the drug was introduced into the culture. For the cometabolic degradation of ibuprofen, 1000 mg L^−1^ glucose, 282 mg L^−1^ phenol, 432 mg L^−1^ benzoate and 417 mg L^−1^ 4-hydroxybenzoate were used as growth substrates. After complete degradation of the suitable growth substrate, a successive dose of the growth substrate was introduced into the culture. All cultures were grown in triplicate and were incubated with shaking at 30 °C for 14 days. Bacterial growth was determined by optical density at *λ* = 600 nm (OD_600_).

Additionally, two control cultures (250 mL each) were prepared: an uninoculated control consisting of a mineral salts medium and a heat-killed control consisting of a mineral salts medium and bacterial cells destroyed by autoclaving. The heat-killed control was used to check the possible adsorption of ibuprofen on fragments of bacterial cells that may result in the decrease of the drug concentration. The optical density of the heat-killed control was the same as for the examined cultures.

### Determination of kinetic parameters of ibuprofen degradation

To study the degradation kinetics, pure cultures of bacterial strain were separately inoculated in a series of 500-mL Erlenmeyer flasks containing 250 mL of the mineral salts medium (Greń et al. 2010) supplemented with ibuprofen at initial concentrations of 1, 3, 5, 7 and 9 mg L^−1^ to a final optical density of 0.8 or 0.1 (OD_600_) in monosubstrate or cometabolic systems, respectively. Based on the results of preliminary studies (unpublished data), in cometabolic systems, glucose at a final concentration of 1 g L^−1^ was added in a solution as the best co-substrate for bacterial growth during ibuprofen degradation. The flasks were incubated with shaking at 30 °C. Samples were taken every 3 h from the monosubstrate and every 2 h from the cometabolic cultures. Growth of the strain was monitored by the optical densities at 600 nm, and the degradation kinetics was studied by the measurements of the residual concentration of ibuprofen in the medium. The concentration of glucose (in cometabolic cultures) was also determined.

The equation by which the death rate in the monosubstrate culture was evaluated according to the Gompertz model is formulated below:

$$ y={\mathrm{y}}^0+a\times { \exp}^{b\times C}, $$

where *y* is the death rate (mg mL^−1^ h^−1^), *a* is the initial death, *b* is the rate of increase in death and *C* is the substrate concentration (mg L^−1^) (Avraam et al. 2013; Casolari 1988; Wu et al. 2008). Kinetic constants were estimated with the exponential growth equation using SigmaPlot 12.0 software.

To study the microbial growth in cometabolic cultures, the Andrew model was used, which is given by the following equation:

$$ \mu =\frac{\mu_{\max}\times S}{K_{S\mu }+S+\left(\frac{S^2}{K_i}\right)} $$

where *μ* is the specific growth rate (mg mL^−1^ h^−1^), *μ*_max_ is the maximum specific growth rate (mg mL^−1^ h^−1^), *K*_*sμ*_ is the substrate concentration in which *μ* = 1/2*μ*_max_ (mg L^−1^), *K*_*i*_ is the inhibition constant (mg L^−1^) and *S* is the ibuprofen concentration (mg L^−1^) (Gąszczak et al. 2010). Kinetic constants were estimated by the Levenberg–Marquardt method using Statistica 10.0 software.

The Monod equation, by which the biodegradation of ibuprofen in monosubstrate and cometabolic cultures was evaluated, is formulated below:

$$ q=\frac{q_{\max}\times S}{K_S+S} $$

where *q* is the specific ibuprofen removal rate (mg L^−1^ h^−1^), *q*_max_ is the maximum specific ibuprofen removal rate (mg L^−1^ h^−1^), *K*_s_ is the half-saturation constant (mg L^−1^) and *S* is the ibuprofen concentration (mg L^−1^) (Durruty et al. 2011; Okpokwasili and Nweke 2005). Kinetic constants were estimated using SigmaPlot 12.0 software.

### Determination of substrate concentration

The concentration of ibuprofen and other aromatic compounds was determined with the HPLC technique using the Merck Hitachi HPLC reversed-phase chromatograph equipped with an Ascentis Express ® C18 HPLC Column (100 × 4.6 mm), an Opti-Solw ® EXP pre-column and a UV/VIS DAD detector. The mobile phase consisting of acetonitrile and 1% acetic acid (70:30 *v*/*v*) at a flow rate of 1 mL min^−1^ was used during the analysis of the ibuprofen concentration. For the determination of phenol, benzoate and 4-hydroxybenzoate concentration, the mobile phase consisting of acetonitrile, methanol and 1% acetic acid (20:20:60 *v*/*v*/*v*) at a flow rate of 1 mL min^−1^ was used. The detection wavelengths were set at 263, 272, 272 and 260 nm for ibuprofen, phenol, benzoate and 4-hydroxybenzoate, respectively (Wojcieszyńska et al. 2014).

Ibuprofen and aromatic cosubstrates in supernatants were identified by comparing the HPLC retention time and UV–visible spectra with those of the external standards. The concentration of glucose in the culture supernatant was determined using a colorimetric anthrone method (Gerhardt et al. 1994).

### Bacterial growth inhibition test

To determine the inhibitory effect of ibuprofen on bacterial growth, a pure culture of *B. thuringiensis* B1(2015b) was grown in the nutrient broth supplemented with ibuprofen in the concentration range 0–2.0 mg L^−1^. The initial optical density of each culture was 0.1 (OD_600_). After 24 h incubation with shaking at 30 °C, the optical density of the cultures was measured. The EC_50_ value was estimated using GraphPad PRISM 6.05 software.

### Toxicity bioassays

In order to assess the toxicity of ibuprofen, acute toxicity, chronic toxicity and mutagenicity tests were performed. To assess the acute toxicity of ibuprofen toward various microorganisms, the MARA test was conducted. The assay was performed using ten reference bacterial strains and one strain of yeast, in 96-well microtitre plates in three independent trials. For each plate, both positive (without ibuprofen) and negative (without microorganisms) controls were included. After 18 h of incubation in the dark at 30 °C, the plates were subjected to image analysis. The microbial growth after exposure to the concentration gradient of ibuprofen was determined by a reduction of tetrazolium salt which precipitated at the bottom of the wells. The results were expressed as microbial toxic concentration (MTC) for each microorganism and for the whole test. The MTC value was calculated according to the formula:

$$ MTC={c}_{\min}\times {d}^{\left(\frac{P_{\mathrm{tot}}}{P_o}\right)-1} $$

where *c*_min_ is the lowest concentration in the gradient, *P*_O_ is the pellet size in the control, *d* is the dilution factor and *P*_tot_ is the sum of all pellet sizes across the concentration gradient (Bronowska et al. 2013; Sieroslawska 2014; Wadhia 2008).

The chronic toxicity of ibuprofen was determined with the use of Protoxkit F (MicroBioTest Inc.) and *Tetrahymena thermophila* as the test organism. The test is based on the turnover of the substrate into the ciliate biomass. *T. thermophila* was grown in the presence of the substrate and 0.625, 1.25, 2.5, 5.0 or 10.0 mg L^−1^ ibuprofen at 30 °C in the dark. After 48 h of incubation, optical density at 440 nm was measured. The inhibition of growth of the culture was reflected by the remaining turbidity.

Ames MPF tests (Xenometrix) were performed with *Salmonella typhimurium* TA98 and *S. typhimurium* TA100 according to the manufacturer’s recommendations. Ten millilitres of the growth medium was inoculated with 10 μL of refrozen bacterial strains and incubated for 16 h at 37 °C with shaking at 250 rpm in the presence of 50 μg mL^−1^ ampicillin until culture density reached >2.0 (OD_600_). The obtained cultures were diluted tenfold into the exposure medium, and 240 μL of the mixture was introduced into every well of a 24-well plate. Simultaneously, an appropriate volume of 50 mg mL^−1^ ibuprofen stock solution was introduced into the wells to obtain the final concentrations of 8, 25, 74, 222, 667 and 2000 mg L^−1^ of ibuprofen. Diluted inocula of bacterial strains were negative controls. The positive controls comprised the diluted inoculum and 0.1 μg mL^−1^ 4-nitroquinoline-*N*-oxide or 2.0 μg mL^−1^ 2-nitrofluorene as mutagens. The mutagenicity assays were also conducted in the presence of metabolic activation fraction S9 (rat liver microsomal fraction). The samples were prepared similarly as described above, except that the microsomal fraction was further introduced into each well. Diluted inocula of bacterial strains were negative controls. The positive controls consisted of diluted inocula of bacteria, fraction S9 and 0.5 or 1.25 μg mL^−1^ 2-aminoanthracene for strains TA98 and TA100, respectively.

The 24-well plates were then incubated for 90 min at 37 °C with shaking at 250 rpm, and the cultures were mixed with the indicator medium. Then, 50-μL aliquots of each culture were replica plated into 48 wells of a 384-well plate and incubated at 37 °C for 48 h. The number of positive wells out of 48 wells per replicate and per tested concentration of ibuprofen was compared with the number of spontaneous revertants obtained in the negative control section (Flückiger-Isler et al. 2004).

### Fatty acid extraction and analysis

The fatty acid composition of *B. thuringiensis* B1(2015b) was determined after 18 h of cultivation of the strain on (1) a nutrient broth (control sample), (2) a nutrient broth containing 0.8 g L^−1^ ibuprofen and (3) a nutrient broth containing 2.0 g L^−1^ ibuprofen. Bacteria were harvested by centrifugation (8000×*g*) at 4 °C for 20 min. The cell pellets were washed twice with 0.85% NaCl to remove the residue of the culture medium. The fatty acid isolation and identification were conducted by the MIDI–MIS method (Sasser 1990). Analysis of the fatty acid methyl esters (FAMEs) was performed using an HP 5890 gas chromatograph (Hewlett Packard, Rolling Meadows, IL, USA) equipped with an HP 25 m × 0.2 mm cross-linked methyl-silicone capillary column. The initial oven temperature was 170 °C. Then, it was increased to 260 °C at 5 °C min^−1^ and then to 320 °C at 40 °C min^−1^, and was held constant for 1.5 min. Helium was used as the carrier gas. FAMEs were identified using Sherlock software (TSBA library, version 3.9, Microbial ID, Newark, NJ, USA) based on the actual calibration retention times run prior to sample analysis.

The results were evaluated by analysis of variance. Three replicates of data obtained from each treatment were analysed statistically by one-way ANOVA. The statistical significance (*p* < 0.05) of the differences was assessed by a post hoc comparison of the means using the least significant difference (LSD) test. The FAMEs profiles were also subjected to principal component analysis (PCA). This was performed based on the average values of three replicates. All analyses were performed using the Statistica 12.0 PL software package.

### Preparation of cell extracts

The cell-free extracts were prepared from the *B. thuringiensis* B1(2015b) cultures grown to the OD_600_ <0.8. The crude extracts were isolated from monosubstrate cultures with ibuprofen, glucose, phenol, benzoate or 4-hydoxybenzoate as a sole carbon source, and from cometabolic cultures with ibuprofen as a cometabolite and glucose, phenol, benzoate or 4-hydroxybenzoate as a growth substrate. The bacterial cells were harvested by centrifugation (4500×*g*) for 15 min at 4 °C. The obtained pellets were washed with 50 mM phosphate buffer, pH 7.0, and resuspended in the same buffer. Then, the whole-cell suspensions were homogenised by sonication (six times for 15 s) and subjected to centrifugation at 9000×*g* for 30 min at 4 °C. Clear supernatants were used as crude cell extracts for the enzyme assays.

### Enzyme assays

Phenol monooxygenase activity was determined spectrophotometrically by measuring NADH oxidation (*ε*_340_ = 6220 M^−1^ cm^−1^) (Divari et al. 2003). The activity of catechol 1,2-dioxygenase was measured spectrophotometrically by the formation of *cis*,*cis*-muconic acid at 260 nm (*ε*_260_ = 16,800 M^−1^ cm^−1^). In order to determine catechol 2,3-dioxygenase activity, the formation of 2-hydroxymuconic semialdehyde was measured at 375 nm (*ε*_375_ = 36,000 M^−1^ cm^−1^) (Wojcieszyńska et al. 2011a). The activity of protocatechuate 3,4-dioxygenase was assayed by measuring the oxygen consumption rate (Hou et al. 1976). The activity of protocatechuate 4,5-dioxygenase was measured spectrophotometrically by the formation of 2-hydroxy-4-carboxymuconic semialdehyde at 410 nm (*ε*_410_ = 9700 M^−1^ cm^−1^) (Wojcieszyńska et al. 2011a). One unit of enzyme activity was defined as the amount of enzyme required to generate 1 μmol of product per minute. Protein concentration in the crude extract was determined by the Bradford method using bovine serum albumin as a standard (Wojcieszyńska et al. 2011a).

## Results and discussion

### Biodegradation studies

**Fig. 1:**
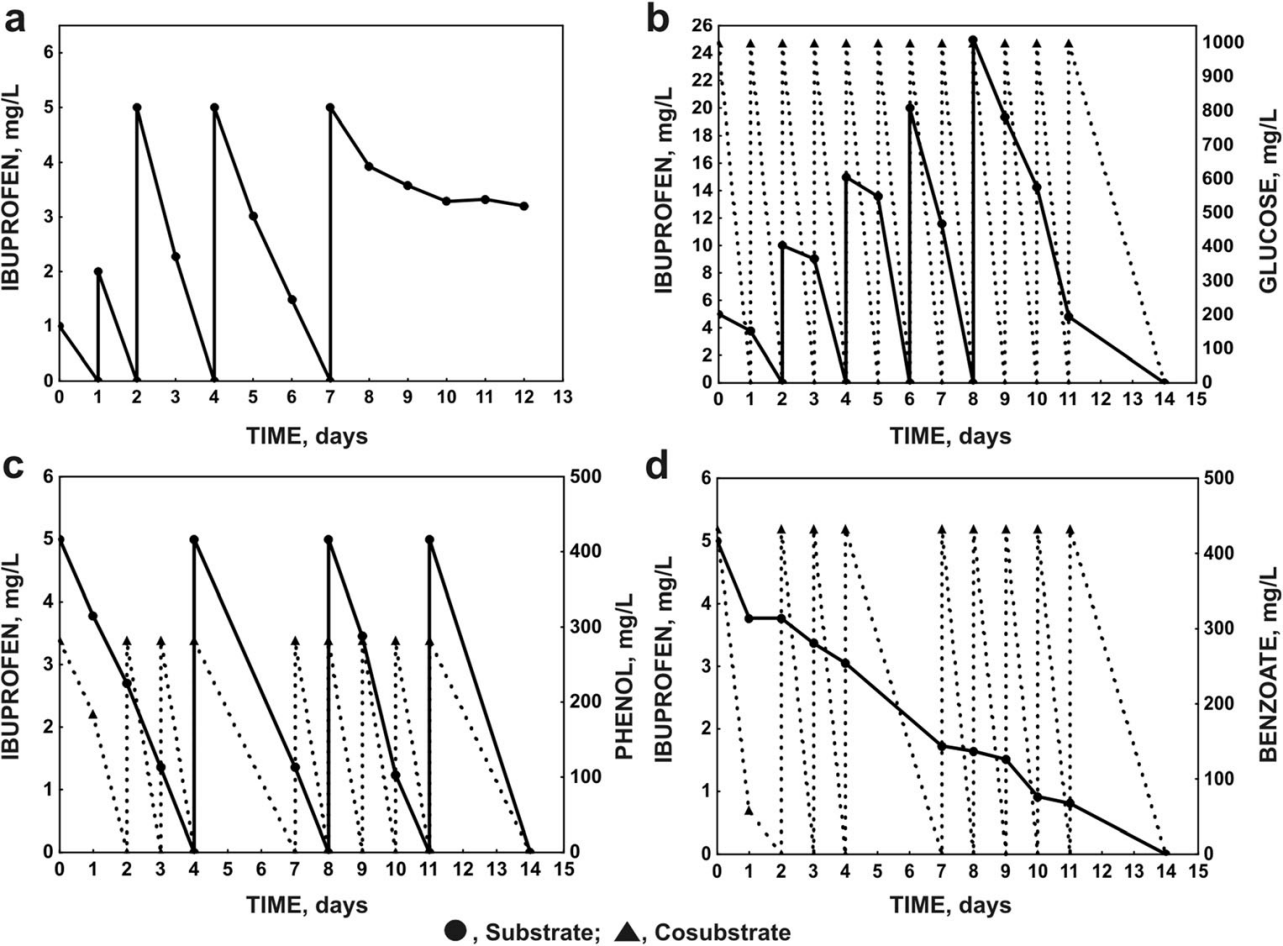
Biodegradation of ibuprofen by *Bacillus thuringiensis* B1(2015b) in monosubstrate (**a**) and cometabolic systems with glucose (**b**), phenol (**c**) and benzoate (**d**) as growth substrates

*Bacillus* species belong to Gram-positive bacteria, which are known for their tolerance to various toxic compounds. This results mainly from the structure of their cellular membranes as well as from their ability to synthesise surface-active agents and specific enzymes (Satchanska et al. 2006; Solyanikova et al. 2014; Swaathy et al. 2014; Trivedi et al. 2011). For this study, *B. thuringiensis* B1(2015b) isolated from the soil at the Chemical Factory “Organika-Azot” in Jaworzno and able to degrade ibuprofen and naproxen was used (Marchlewicz et al. 2016). The adaptation of *B. thuringiensis* B1(2015b) strain to increasing concentrations of ibuprofen showed its ability to degrade up to 5 mg L^−1^ of this drug. However, in the presence of ibuprofen as a sole carbon and energy source, growth of the culture was not observed, and consequently, the B1(2015b) strain lost its degradation activity (Fig. 1a). It is generally known that the introduction of an additional carbon source to the culture may enhance the metabolism of xenobiotics. For example, Quintana et al. (2005) showed that the degradation of 5 mg L^−1^ ibuprofen by activated sludge lasted for over 28 days, while in the presence of powder milk as an additional carbon source, complete degradation of the introduced dose of ibuprofen was observed after 22 days. In the studies on the cometabolic degradation of ibuprofen, glucose was chosen as an easily assimilated growth substrate. Simultaneously, as growth substrates, phenol, benzoate and 4-hydroxybenzoate were used. These aromatic compounds are substrates of similar structure to ibuprofen and are known to induce synthesis of the enzymes engaged in aromatic ring fission (Wojcieszyńska et al. 2014). The highest rate of ibuprofen degradation was observed in the presence glucose as the growth substrate. Under these conditions, B1(2015b) was able to degrade completely up to 25 mg L^−1^ of ibuprofen (Fig. 1b). In turn, the addition of aromatic compounds as growth substrates resulted in the decreased ability of strain B1(2015b) to degrade ibuprofen. In the presence of these compounds, B1(2015b) degraded only up to 5 mg L^−1^ of ibuprofen (Fig. 1c, d). This may result from the competition between aromatic growth substrates and ibuprofen for the active site of enzymes involved in aromatic ring degradation (Wang et al. 2015). Particularly, in the presence of benzoate or 4-hydroxybenzoate as a carbon source, degradation of ibuprofen was ineffective. In the presence of benzoate, complete degradation of 5 mg L^−1^ of ibuprofen was observed after 14 days of incubation (Fig. 1d). In the presence of 4-hydroxybenzoate after 4 days of incubation, darkening of the culture was observed. At the same time, a 40.8% loss of ibuprofen and a 100% loss of 4-hydroxybenzoate were determined (data not shown). This may suggest condensation of ibuprofen and 4-hydroxybenzoate, as carboxylic acids are known to initiate this kind of reaction (Stebbins et al. 2015; Wasiniak and Lukaszewicz 2010). Additionally, the presence of the hydroxy group in the *para* position of 4-hydroxybenzoate facilitates the condensation of these compounds.

In the presence of the growth substrates, the activity of enzymes engaged in aromatic compound metabolism was observed. The activity of monooxygenase and catechol 1,2-dioxygenase was observed in the crude extracts from each monosubstrate culture (Table 1). The higher activity of monooxygenase in the presence of ibuprofen, phenol and benzoate reveals hydroxylation, the first step of aromatic compound degradation (Wojcieszyńska et al. 2011b), while the activity of catechol 1,2-dioxygenase indicates *ortho* cleavage of the aromatic ring of these compounds (Guzik et al. 2013). In the presence of 4-hydroxybenzoate as an inductor, the activity of monooxygenase and catechol 1,2-dioxygenase was significantly lower than in the control culture with glucose (Table 1). In turn, in the presence of benzoate and 4-hydroxybenzoate, high activity of protocatechuate 3,4-dioxygenase was observed (Table 1). This suggests the engagement of this enzyme in the degradation of the aromatic ring of carboxylic acid. Additionally, the high activity of this enzyme results from its induction by the product of the ring cleavage—3-carboxy-*cis*,*cis*-muconic acid (Tropel and van der Meer 2004).

**Table 1:** Specific activity of enzymes in monosubstrate cultures

Enzyme	Specific enzyme activity (U mg^−1^ protein)				
	IBU	GLC	PH	BS	4-HB
Monooxygenase	20.64 ± 1.05	19.68 ± 1.50	21.29 ± 1.72	20.27 ± 0.79	8.80 ± 0.98
Catechol 1,2-dioxygenase	56.29 ± 0.72	41.31 ± 3.77	199.10 ± 10.24	35.16 ± 5.21	16.60 ± 1.82
Catechol 2,3-dioxygenase	0.0 ± 0.0	0.0 ± 0.0	0.0 ± 0.0	0.0 ± 0.0	0.0 ± 0.0
Protocatechuate 3,4-dioxygenase	0.0 ± 0.0	0.0 ± 0.0	0.0 ± 0.0	392.71 ± 0.0	7724.2 ± 0.0
Protocatechuate 4,5-dioxygenase	0.0 ± 0.0	0.0 ± 0.0	0.0 ± 0.0	5.30 ± 1.07	0.0 ± 0.0

*IBU* ibuprofen, *GLC* glucose, *PH* phenol, *BS* benzoate, *4-HB* 4-hydroxybenzoate

Until now, the activity of enzymes engaged in degradation of aromatic compounds in the presence of ibuprofen was not described. In this study, in the cometabolic systems with ibuprofen and benzoate or 4-hydroxybenzoate as a growth substrate, the inhibition of protocatechuate 3,4-dioxygenase was observed (Table 2). It may result from the fact that ibuprofen is a competitive inhibitor of cyclooxygenase-2 and the arachidonic acid oxidative enzyme (Prusakiewicz et al. 2009), which, similarly to protocatechuate 3,4-dioxygenase, belong to the family of non-heme oxygenases (Abu-Omar et al. 2005; Guzik et al. 2013). Moreover, active sites of both cyclooxygenase-2 and protocatechuate 3,4-dioxygenase are long hydrophobic channels with a number of basic amino acid residues (e.g. arginine). It is suggested that the carboxylic group of ibuprofen forms a salt bridge with the guanidinium group of arginine and blocks the entrance into the active site of cyclooxygenase (Dannhardt and Kiefer 2001). We assume that ibuprofen interacts with arginine residues of the hydrophobic channel of protocatechuate 3,4-dioxygenase in the same manner and inhibits the enzyme. The lack of inhibitory effect of ibuprofen on catechol 1,2-dioxygenase, which also belongs to the family of non-heme oxygenases, may result from the distinct structure of the entrance to the active site of this enzyme (Guzik et al. 2013).

**Table 2:** Specific activity of enzymes in cometabolic cultures

Enzyme	Specific enzyme activity (U mg^−1^ protein)			
	IBU + GLC	IBU + PH	IBU + BS	IBU + 4-HB
Monooxygenase	41.27 ± 1.34	17.92 ± 0.43	18.81 ± 1.91	39.43 ± 0.52
Catechol 1,2-dioxygenase	44.22 ± 1.15	97.40 ± 1.82	40.37 ± 5.58	68.17 ± 4.46
Catechol 2,3-dioxygenase	0.0 ± 0.0	0.0 ± 0.0	0.0 ± 0.0	0.0 ± 0.0
Protocatechuate 3,4-dioxygenase	0.0 ± 0.0	0.0 ± 0.0	0.0 ± 0.0	2212.90 ± 136.07
Protocatechuate 4,5-dioxygenase	0.0 ± 0.0	0.0 ± 0.0	0.0 ± 0.0	10.89 ± 0.50

*IBU* ibuprofen, *GLC* glucose, *PH* phenol, *BS* benzoate, *4-HB* 4-hydroxybenzoate

### Kinetic models of ibuprofen degradation

**Fig. 2:**
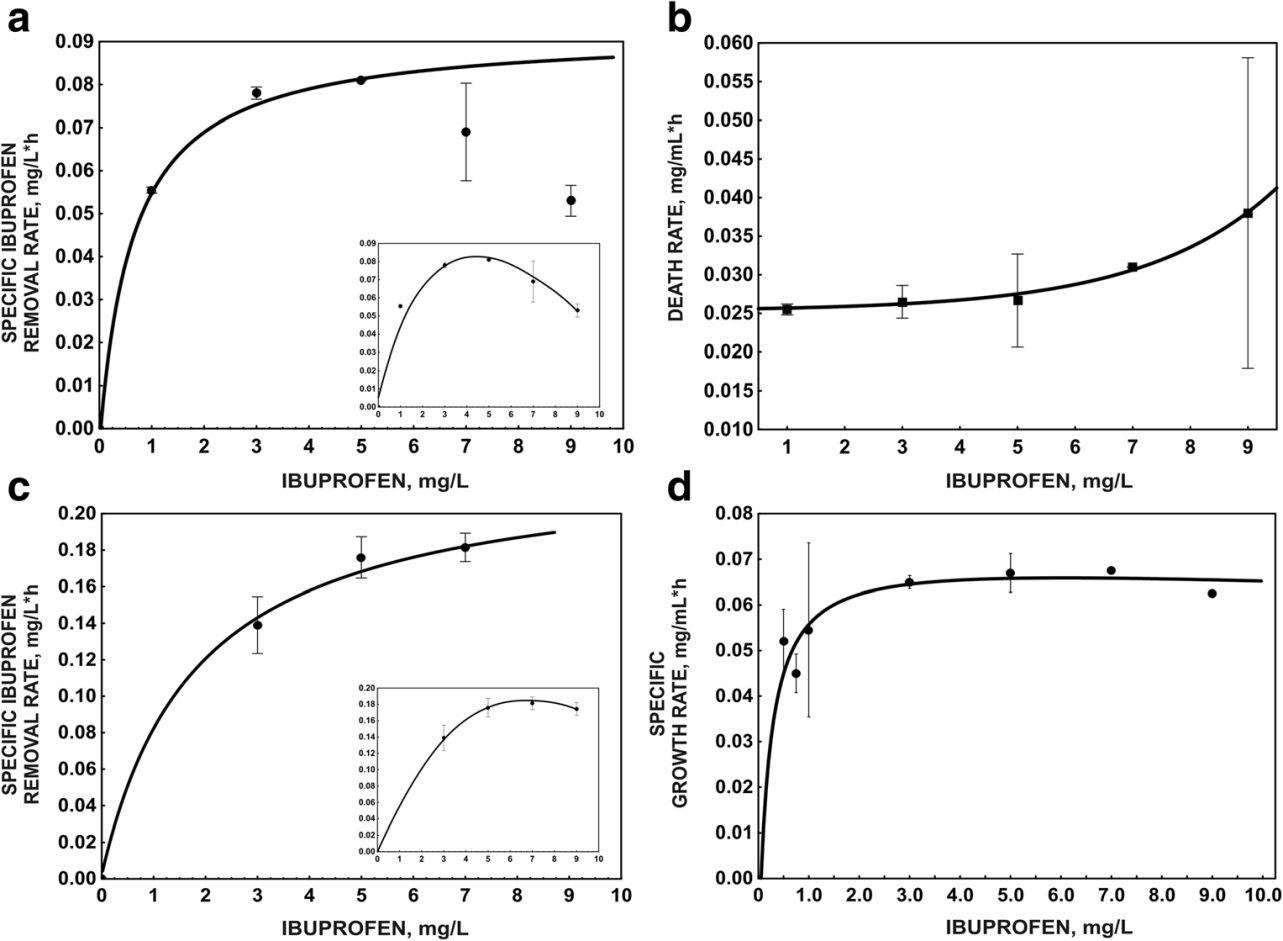
Kinetic models of ibuprofen degradation (**a**) and bacterial survival (**b**) in the monosubstrate system and ibuprofen degradation (**c**) and bacterial growth (**d**) in the cometabolic system with glucose as a growth substrate. The *data points* represent the average of three independent experiments

Despite the large amount of information about degradation of ibuprofen by the pure bacterial culture or microbial consortia, the kinetic model of ibuprofen degradation by the pure bacterial strain has not been described. For that reason, kinetic analysis of ibuprofen degradation by *B. thuringiensis* B1(2015b) was performed in monosubstrate or cometabolic systems with glucose as a growth substrate. The dependence of the specific ibuprofen removal rate on the ibuprofen concentration in monosubstrate and cometabolic systems is shown in panels a and c of Fig. 2, respectively. The half-saturation constant (*K*_s_) and the maximum specific ibuprofen removal rate (*q*_max_) were higher for the cometabolic system (*K*_s_ = 2.12 ± 0.56 mg L^−1^; *q*_max_ = 0.24 ± 0.02 mg L^−1^ h^−1^) than for the monosubstrate system (*K*_s_ = 0.68 ± 0.08 mg L^−1^; *q*_max_ = 0.09 ± 0.00 mg L^−1^ h^−1^). The half-saturation constant expresses a bacterial affinity for a substrate (Kim et al. 2003). Therefore, the lower *K*_s_ obtained for the monosubstrate system suggests that *B. thuringiensis* B1(2015b) is able to degrade ibuprofen faster at lower concentrations. The obtained results also showed that the introduction of an additional carbon source has a positive effect on ibuprofen degradation by *B. thuringiensis* B1(2015b). The increased degradation of a xenobiotic compound in the presence of an additional growth substrate was observed by Durruty et al. (2011). In their work, the simultaneous degradation of various chlorophenols was a key factor improving the degradation of pentachlorophenol (Durruty et al. 2011). The inhibitory effect of the substrate on its degradation is frequently observed. For example, Sinha et al. (2011) described the inhibition of degradation by the substrate during cultivation of *Rhodococcus* sp. RSP8 in the presence of phenol or *p*-chlorophenol. During our studies on degradation of ibuprofen by strain B1(2015b) in both monosubstrate and cometabolic cultures, the inhibition of this process by the substrate was also observed. In the monosubstrate system, inhibition was observed at a lower concentration of ibuprofen (6 mg L^−1^) than in the cometabolic system (9 mg L^−1^) (Fig. 2a, c). This was likely caused by bacterial cell death observed in the monosubstrate system (Fig. 2b). The initial death rate of 0.0003 ± 0.0002 increased to 0.44 ± 0.07. The kinetics of microbial death indicates that ibuprofen is an insufficient carbon source for bacteria. In turn, in the cometabolic system, the growth of bacterial cells was observed (Fig. 2d). In the presence of glucose as an additional carbon source, the maximum specific growth rate was 0.07 ± 0.01 mg mL^−1^ h^−1^, *K*_s*μ*_ was 0.27 ± 0.15 mg L^−1^ and *K*_*i*_ was 137.16 mg L^−1^. The increase of the maximum specific ibuprofen removal rate in the cometabolic system results from the high microbial biomass. In turn, the high half-saturation constant observed under cometabolic conditions may be connected with the competition between enzymes involved in ibuprofen degradation and those engaged in glucose metabolism for the cofactors.

### Toxicity of ibuprofen

**Fig. 3:**
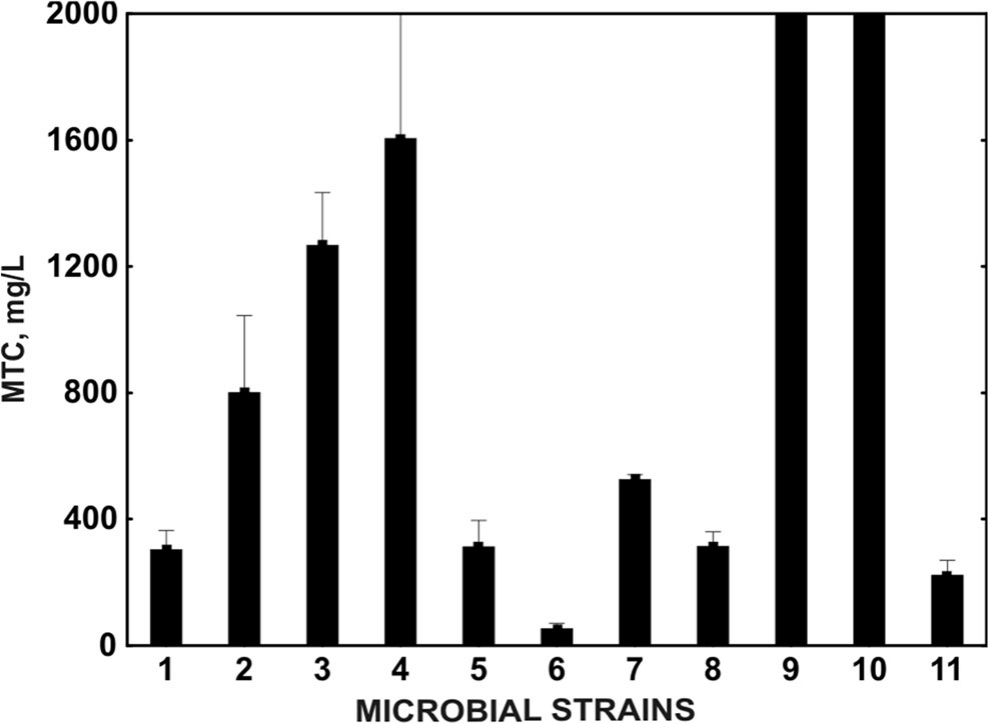
Mean MTC values in the MARA test; ibuprofen exposition ranged between 63 and 2000 mg L^−1^. Microorganisms used in the MARA test: *1 Microbacterium* sp. G(+), *2 Brevundimonas diminuta* G(−), *3 Citrobacter freudii* G(−), *4 Comamonas testosteroni* G(−), *5 Enterococcus casseliflavus* G(+), *6 Delftia acidovorans* G(−), *7 Kurthia gibsonii* G(+), *8 Staphylococcus warneri* G(+), *9 Pseudomonas aurantiaca* G(−), *10 Serratia rubidaea* G(−), *11 Pichia anomala* (yeast)

**Fig. 4:**
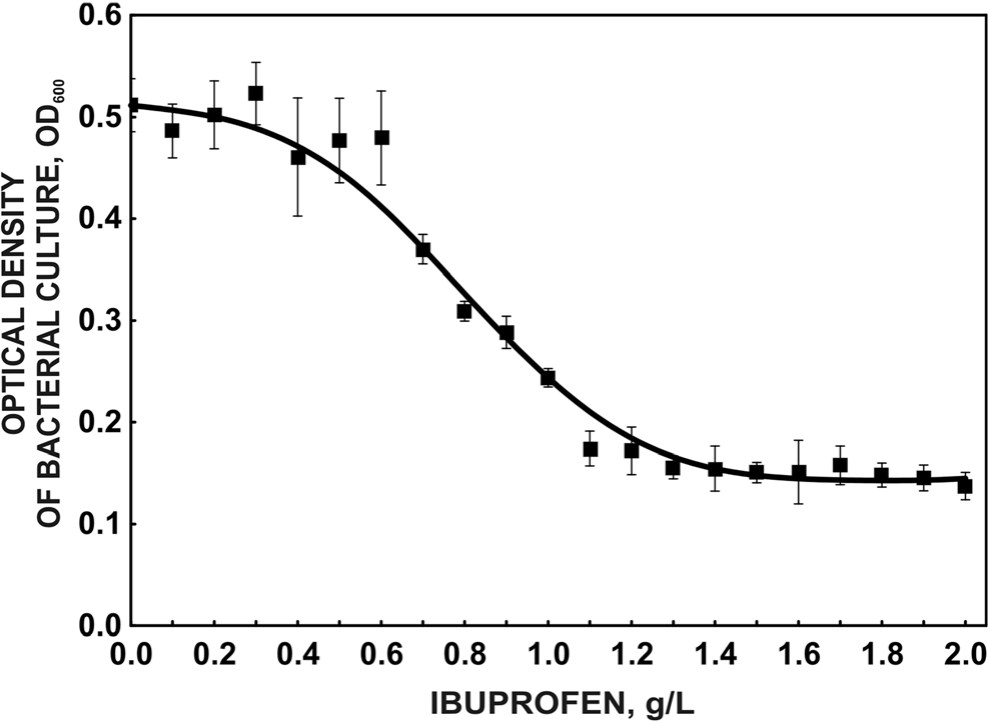
Inhibition of *Bacillus thuringiensis* B1(2015b) growth in the presence of ibuprofen at various concentrations

Ibuprofen is known to induce changes in the functioning of several physiological systems of different organisms, probably through the inhibition of prostaglandin synthesis. For example, in mussels, *Mytilus galloprovincialis*, in the presence of ibuprofen, reproductive fitness impairment was observed (Gonzales-Rey and Bebianno 2012). Evaluation of the acute toxicity of ibuprofen showed different responses of tested strains. The most sensitive microorganism was *Delftia acidovorans*. The MTC for this strain was 311.5 ± 84.15 mg L^−1^, whereas the most durable strains were *Pseudomonas aurantiaca* and *Serratia rubidaea* (MTC above 2000 mg L^−1^) (Fig. 3). The mean value of the microbial toxic concentration MTC_avg_, which is the equivalent of EC_50_, was 545.50 ± 7.78 mg L^−1^. EC_50_ estimated for *B. thuringiensis* B1(2015b) grown in a nutrient broth supplemented with ibuprofen at a concentration range from 0 to 2.0 mg L^−1^ was 809.3 mg L^−1^ (Fig. 4). According to EU Directive 93/67/EEC, chemical substances are classified into different risk classes on the basis of the lowest measured EC_50_. Compounds with an EC_50_ ≤ 1 mg L^−1^ (class 1) are considered to be very toxic to aquatic organisms. Substances with an EC_50_ above 1 mg L^−1^ and below 10 mg L^−1^ are toxic to aquatic organisms, and those with an EC_50_ above 10 mg L^−1^ but below 100 mg L^−1^ are considered as harmful to aquatic organisms. Substances with an EC_50_ > 100 mg L^−1^ are recognised as nontoxic (Cleuvers 2004; Ortiz de Garcia et al. 2014). The determination of the toxic effect of ibuprofen on various organisms with the use of MARA and inhibition growth (Protoxkit F) tests showed that ibuprofen is non-toxic to aquatic organisms (Fig. 3 and Fig. 4). Moreover, the very low (*a* = 0.0003) initial death parameter, which determines the death rate, estimated for *B. thuringiensis* B1(2015b) grown in the presence of different concentrations of ibuprofen also indicates the low toxicity of ibuprofen. Therefore, various coincidence parameters (EC_50_ and *a*) may be interchangeably used for the evaluation of the toxicity of xenobiotics.

**Fig. 5:**
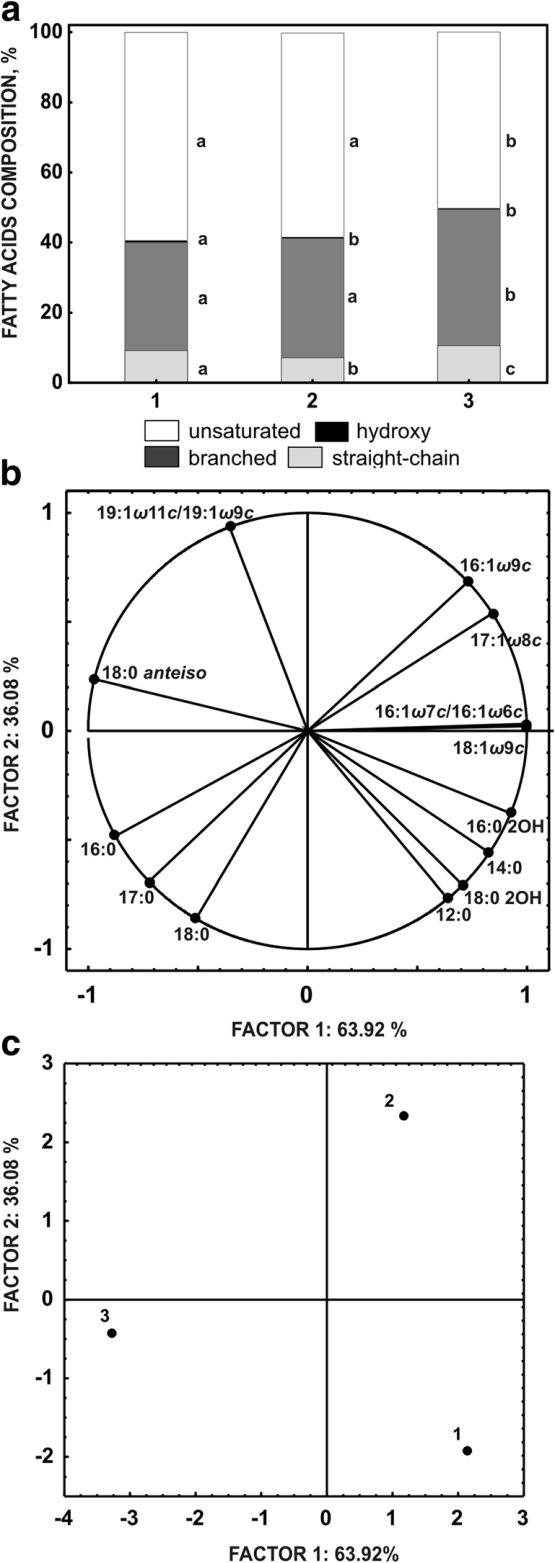
**a** Relative proportions of fatty acids in *Bacillus thuringiensis* B1(2015b) grown on nutrient broth (*1*), nutrient broth supplemented with 0.8 g L^−1^ ibuprofen (*2*) and nutrient broth supplemented with 2.0 g L^−1^ ibuprofen (*3*). Class of hydroxyl fatty acids additionally contains branched hydroxyl fatty acids. *Different letters* indicate a statistically significant difference between fatty acid groups. **b** Configuration of points representing the fatty acids in the system of the first two factorial axes (principal components). **c** Principal component analysis of fatty acid proportion in *Bacillus thuringiensis* B1(2015b) grown on nutrient broth with the addition of ibuprofen

Toxic compounds can interact with the cell membranes of bacteria and influence their integrity and fluidity (Murínova and Dercová 2013; Segura et al. 2010). Ibuprofen as a stressor, due to its amphipathic features, shows high affinity to the phospholipid bilayer of bacteria. Therefore, it was very interesting to evaluate whether the high resistance of *B. thuringiensis* B1(2015b) to ibuprofen was connected with changes in the membrane fatty acid composition. The whole cell-derived fatty acid profiles of strain B1(2015) grown in a nutrient broth supplemented with 0.8 or 2.0 g L^−1^ ibuprofen and in nutrient broth as the control were compared. 0.8 g L^−1^ ibuprofen was used as the concentration equal to the EC_50_ value, while 2.0 mg L^−1^ ibuprofen was the concentration at which maximal growth inhibition was observed. Identified fatty acids were divided into two groups: saturated and unsaturated fatty acids. Saturated fatty acids were additionally divided into straight-chain, branched and hydroxy fatty acids (Fig. 5a). The obtained results clearly showed that both the presence of ibuprofen and its concentration alter the fatty acid composition of *B. thuringiensis* B1(2015b). The FAMEs profile of strain B1(2015b) grown in the presence of 0.8 g L^−1^ ibuprofen was distinct from others regarding factor 2. However, the contribution of fatty acids discriminating this profile (16:1 ω9c, 17:1 ω8c and 19:1 ω11c) was low (Table 3). In the presence of 2.0 g L^−1^ ibuprofen, a high content of branched fatty acids and a low content of unsaturated fatty acids were identified (Fig. 5a). Principal component analysis of the identified fatty acids revealed that the crucial branched fatty acid was 18:0 *anteiso*, and the unsaturated fatty acid was 18:1 ω9*c* (Fig. 5b, c). The increased content of branched fatty acids together with the high content of long-chain fatty acids in bacterial membranes modulates their fluidity through the increase of the phase transition temperature, which causes the decrease of membrane permeability (Lindström et al. 2006). The high content of branched fatty acids determined in *B. thuringiensis* B1(2015b) grown in the presence of ibuprofen at a concentration of 2.0 g L^−1^ may result from their synthesis de novo (López-Lara and Geiger 2010). The increased contribution of *anteiso* branched fatty acids in Gram-positive *Arthrobacter chlorophenolicus* A6 grown in the presence of phenol, 4-chlorophenol and 4-nitrophenol was also observed. Additionally, these aromatic compounds altered the ratio of *anteiso/iso* fatty acids (Unell et al. 2007). In our studies the *iso* branched fatty acids were not detected. The changes in the ratio of branched and unsaturated fatty acids of strain B1(2015b) may increase the tightness of bacterial membranes, which can in turn be the adaptive feature of the bacterium to grow in the presence of ibuprofen.

**Table 3:** Percentage of total fatty acids from *Bacillus thuringiensis* B1 grown on nutrient broth (1), nutrient broth supplemented with 0.8 g L^−1^ ibuprofen (2) and nutrient broth supplemented with 2.0 g L^−1^ ibuprofen (3). Data represent the average of three independent trials ± standard deviation

Fatty acids	% of total fatty acids		
	1	2	3
Saturated			
12:0	0.14 ± 0.03	0.00 ± 0.00	0.00 ± 0.00
14:0	1.05 ± 0.00	0.66 ± 0.07	0.51 ± 0.06
16:0	6.76 ± 0.06	5.79 ± 0.09	8.68 ± 0.34
16:0 2OH	0.34 ± 0.05	0.25 ± 0.07	0.17 ± 0.00
17:0	0.11 ± 0.00	0.06 ± 0.08	0.15 ± 0.03
18:0	1.14 ± 0.08	0.62 ± 0.05	1.26 ± 0.15
18:0 2OH	0.29 ± 0.04	0.19 ± 0.00	0.17 ± 0.02
18:0 anteiso	30.80 ± 1.55	34.01 ± 0.55	38.87 ± 1.55
Unsaturated			
16:1 ω7c	1.71 ± 0.22	1.61 ± 0.07	1.06 ± 0.13
16:1 ω9c	0.09 ± 0.00	0.20 ± 0.01	0.00 ± 0.00
17:1 ω8c	0.65 ± 0.00	0.72 ± 0.00	0.54 ± 0.00
18:1 ω9c	56.88 ± 1.09	55.65 ± 0.43	48.48 ± 1.59
19:1 ω11c	0.07 ± 0.09	0.15 ± 0.02	0.12 ± 0.01
20:1 ω9c	0.00 ± 0.00	0.10 ± 0.14	0.00 ± 0.00
Sat./unsat. ratio	0.68 ± 0.04	0.71 ± 0.01	0.99 ± 0.07

*ω* methyl end of fatty acid; *c cis* configuration of the double bond; *–OH* indicates the position of the hydroxyl group from the acid end; *iso*, *anteiso* branched fatty acids

The results of the acute toxicity tests of ibuprofen do not rule out its chronic toxic effect on organisms. It is suggested that the chronic effects of ibuprofen on organisms is connected with its influence on eicosanoid pathways, which are similar in non-insect invertebrates and vertebrates (Pounds et al. 2008). The effects of chronic exposure of *Menidia beryllina* on ibuprofen were described by Jeffries et al. (2015). In our studies, the chronic effect of ibuprofen to ciliate *T. thermophila* was determined with the use of the Protoxkit F test. The results of toxicity tests showed that ibuprofen at the two highest concentrations causes the inhibition of *T. thermophila* growth of approximately 38% in comparison with the control (Table 4). The EC_50_ estimated with the use of Protoxkit F was 17.882 mg L^−1^. Longer exposition of the ciliate to ibuprofen at a lower concentration revealed its toxic effects on this organism.

**Table 4:** Optical density at 440 nm and estimation of EC_50_ with Protoxkit F

Ibuprofen concentration, mg L^−1^	OD_T0_ ± SD	OD_T48_ ± SD	% inhibition	EC_50_, mg L^−1^
0	0.503 ± 0.037	0.293 ± 0.022	0.000	17.882
0.625	0.531 ± 0.005	0.331 ± 0.014	5.000	
1.25	0.521 ± 0.016	0.335 ± 0.033	11.667	
2.5	0.541 ± 0.015	0.384 ± 0.028	25.238	
5.0	0.521 ± 0.008	0.392 ± 0.018	38.571	
10.0	0.530 ± 0.012	0.399 ± 0.006	37.857	

The mutagenic potential of ibuprofen was also studied. The results of the mutagenicity assays presented in Table 5 indicate that no significant increase in the number of revertant colonies was observed. According to the EPA and GenPharmTox guidelines, the over twofold increase in mutant frequency in the same experiment indicates the mutagenic potential of the compound (Lah et al. 2005). In our study, similarly to Philipose et al. (1997), no mutagenic activity of ibuprofen was demonstrated in tests with *Salmonella* strains (Table 5).

**Table 5:** Mutagenic activity of ibuprofen expressed as the mean and standard deviation of the number of revertants of strains TA98 and TA100 treated with various concentrations of ibuprofen without, or in the presence of, the metabolic activation system (S9)

Dose level (mg L^−1^)	Without metabolic activation system					
	TA98			TA100		
	Number of revertants/plate	Fold increase (over the baseline 2.15)	*t* test *p* value	Number of revertants/plate	Fold increase (over the baseline 17.35)	*t* test *p* value
0	1.33 ± 0.82	–	–	12.50 ± 4.85	–	–
8	1.33 ± 1.53	0.62	0.50	11.33 ± 4.93	0.65	0.37
25	1.00 ± 1.00	0.47	0.30	15.00 ± 2.65	0.87	0.22
74	2.00 ± 0.00	0.93	0.11	12.67 ± 1.16	0.73	0.48
222	1.33 ± 0.58	0.62	0.50	14.00 ± 2.00	0.81	0.32
667	3.00 ± 2.00	1.40	0.05	13.67 ± 5.03	0.79	0.37
2000	0.67 ± 0.58	0.31	0.13	17.00 ± 1.00	0.98	0.08
Dose level (mg L^−1^)	With metabolic activation system					
	TA98			TA100		
	Number of revertants/plate	Fold increase (over the baseline 3.00)	*t* test *p* value	Number of revertants/plate	Fold increase (over the baseline 19.80)	*t* test *p* value
0	1.17 ± 1.84	–	–	14.00 ± 5.80	–	–
8	1.33 ± 1.16	0.44	0.45	12.67 ± 2.08	0.64	0.36
25	3.00 ± 1.73	1.00	0.10	10.00 ± 4.00	0.51	0.16
74	1.33 ± 0.58	0.44	0.44	12.67 ± 4.16	0.64	0.37
222	1.00 ± 1.00	0.33	0.45	14.00 ± 2.00	0.71	0.50
667	2.00 ± 0.00	0.67	0.24	16.33 ± 1.53	0.83	0.26
2000	1.33 ± 0.58	0.44	0.44	16.33 ± 1.16	0.83	0.26

The performed studies showed the low toxicity of ibuprofen toward tested organisms and no mutagenic activity of this drug. However, ibuprofen may indirectly influence processes regulated by prostaglandins, which are the key regulators of reproductive processes (ovulation, implantation, menstruation), and inflammation processes, pain sensation, sleep and allergy (Jabbour and Sales 2004; Zhou et al. 2010). The inhibition of prostaglandin synthetase activity by ibuprofen may disturb the above-mentioned processes in non-insect invertebrates and vertebrates.

In conclusion, *B. thuringiensis* B1(2015b) is able to degrade ibuprofen both in monosubstrate and cometabolic systems. However, ibuprofen is not a sufficient carbon source for this strain. The effective degradation of this drug occurs in the presence of glucose. Toxicity studies showed that ibuprofen has an EC_50_ for the B1(2015b) strain of 809.3 mg L^−1^, and it is higher than the microbial toxic concentration MTC_avg_ (545.50 ± 7.78 mg L^−1^). This indicates that the examined strain is resistant to ibuprofen. The above-mentioned features of *B. thuringiensis* B1(2015b) suggest the possibility of its use in bioremediation processes.
